# On the Coexistence of Monitoring and Dampening of Pain Perception During the Fight-or-Flight Response

**DOI:** 10.7759/cureus.111311

**Published:** 2026-06-22

**Authors:** Robert B Raffa, Wolfgang Fink, Anuj D Tripathi

**Affiliations:** 1 Pharmacy, Temple University, Philadelphia, USA; 2 Visual and Autonomous Exploration Systems Research Laboratory, The University of Arizona, Tucson, USA

**Keywords:** ascending and descending pain pathways, control system, feedback loop, fight-or-flight, lotka-volterra

## Abstract

Pain-stimulus input is transmitted by mechanisms that respond to real or perceived tissue injury and propagate the information to the spinal cord for reflex withdrawal and to the brain for intentional withdrawal. To be useful, perception of the pain signal needs to be a reliable indicator of the magnitude and duration of the sensory input, i.e., threat, so that appropriate action can be taken, such as fight or flight. Two components of signal transmission are the “ascending pathways,” mediating sensory input → perception, and the “descending pathways,” mediating perception → response. Ascending pathways are often modulated by descending pathways. We previously introduced Lotka-Volterra-style coupling to model ascending and descending pain pathways as a coupled control and feedback loop. Here, we apply the model to examine the value of modulatory feedback to the fight-or-flight response and identify separate response functions that allow for both a quantitative assessment of the immediate threat, i.e., the magnitude of the acute pain signal, and damping of the signal in order to preclude sensory overload leading to counterproductive inaction. As a result of increasing inhibitory modulation that accompanies increasing pain-stimulus magnitude, pain perception rises rapidly as a function of stimulus intensity and then begins to decay as modulation ramps up, but not to zero. Instead, it saturates or plateaus at a value intermediate between zero and peak/maximum perception, at a level that is related to the stimulus intensity. Although the contribution of this work is conceptual and application-focused rather than presenting new mechanistic insights, the observed response functions, i.e., pain perception and modulation, allow for both a quantitative assessment of the immediate threat and damping of the pain signal in order to preclude sensory overload that might lead to counterproductive inaction. Moreover, the fact that plateau pain perception is less than peak/maximum pain perception for respective sensory inputs prevents obscuration of subsequent new, i.e., cumulative, injury.

## Introduction

The concept now known as the fight-or-flight response evolved from Cannon WB’s 1915 description of two “instincts” that occur in animals in response to states of “emotional excitement” [1]. Despite subsequent expanded versions [2], in essence, the fight-or-flight response consists of automatically activated defensive behaviors for dealing with threat [3]. Although typically described as mediated by activation of the sympathetic subdivision of the autonomic nervous system [4], we recently suggested that the sensation of pain should be considered an innate feature of the fight-or-flight response [5] because of its survival value. Indeed, the genetic absence of an ability to sense pain is known to be detrimental to development and survival [6]. In order to have survival value, however, two aspects of pain perception should coexist: (i) detection of pain magnitude and summation of injury to inform the decision whether to fight or flee, and (ii) damping of the signal so that survival-relevant injury signals, i.e., pain, do not overwhelm the ability to assess the progression of the interaction with the threat. That is, the interaction with the threat is not static; it progresses from initial contact to final resolution [2]. For this to occur, pain perception must be both quantitatively and temporally aligned with the extant magnitude of the danger. It must be triggered relatively rapidly upon injury and then attenuated in a timely manner in order to allow for the detection of potential subsequent input signals. However, it cannot be attenuated to zero, because that would preclude monitoring of persisting summated injury.

In order to accomplish the complex demands of pain perception during the fight-or-flight response, there needs to be coordinated input and modulation between the ascending and descending pathways. Peripheral tissue damage gives rise to noxious stimuli that activate detectors, i.e., nociceptors [7,8], which convert chemical signals, e.g., prostaglandin, into an electrochemical signal transmitted via neurons [9] to the dorsal horn of the spinal cord by primary afferents and then by ascending pathways to higher centers where perception occurs [10,11]. The ascending pain stimulus pathways have long been known and characterized [12]; however, appreciation of the important contributions of descending modulating pathways and neurotransmitter systems to the overall phenomenon of pain is more recent [13,14]. The descending pathway involves brainstem and midbrain structures, such as the periaqueductal gray (PAG) and rostral ventromedial medulla (RVM), which receives relay neuronal input from the PAG [15,16]. Diffuse bilateral projections from the RVM terminate at multiple levels in the brainstem and dorsal horn of the spinal cord [17-19], either amplifying or attenuating the incoming sensory input, thereby modulating pain perception [20-22].

We have developed a control-system approach to model normal pain-processing conditions [23]. The model is based on the realization that the ascending and descending pathways cannot function optimally if they act as independent countercurrent pathways. To function in a controlled manner, they should be connected in a coordinated, coupled, and well-controlled feedback loop. This concept, which leads naturally to the extrapolation that pain can therefore be modeled as a control system, was introduced in a previous presentation [24] and recently actualized [23]. We hypothesize that the fight-or-flight response has the ability to transmit both (i) a transitory indication of the magnitude of the immediate injury and (ii) an attenuated, more prolonged monitoring of the cumulative injury.

Our original paper [23] established the theoretical foundations of our control-system model and described its general broad capabilities without presenting a specific application. The current report applies our model to explore a specific biological application, namely, simulation of a fight-or-flight response scenario.

## Materials and methods

To simulate an attack that would elicit a typical fight-or-flight response, e.g., a rapid-onset, short-duration bite or swipe of a paw inducing acute pain, we modeled the event while ignoring delayed secondary events, such as wound infection. The modeling equivalent is a rectangular function for the sensory input, i.e., the pain stimulus, where, in the absence of modulation *M(t)*, pain perception *P(t)* rises rapidly in response to the pain stimulus *S(t)* through ascending pain-transmitting pathways, then returns to baseline once the stimulus has stopped. One might envision a sudden bite by an animal that lasts for seconds and is then released. We add *M(t)* to include the influence of descending pain-modulating, i.e., attenuating, pathways on pain perception *P(t)*. The underlying model equations, derived in detail in Fink W and Raffa RB [23], were numerically solved/integrated using the standard Runge-Kutta-4 method with constant time steps [25], and are:

Sensory input: $S(t) = \begin{cases} 0 & \text{for } t &lt; t_{\mathrm{onset}} \\ S_{\max} & \text{for } t_{\mathrm{onset}} \leq t \leq t_{\mathrm{off}} \\ 0 & \text{for } t > t_{\mathrm{off}} \end{cases}$

Modulation change over time (rate):

$\frac{d M(t)}{dt}=\varepsilon_mP(t)-\beta_mM(t)$

Pain perception change over time (rate) with Lotka-Volterra-style coupling to modulation in the last term:

$\frac{d P(t)}{dt}=\varepsilon_pS(t)-\beta_pP(t)-\alpha_pP(t)M(t)$

MATLAB (MathWorks) was used for simulations and analyses.

## Results

**Figure 1:**
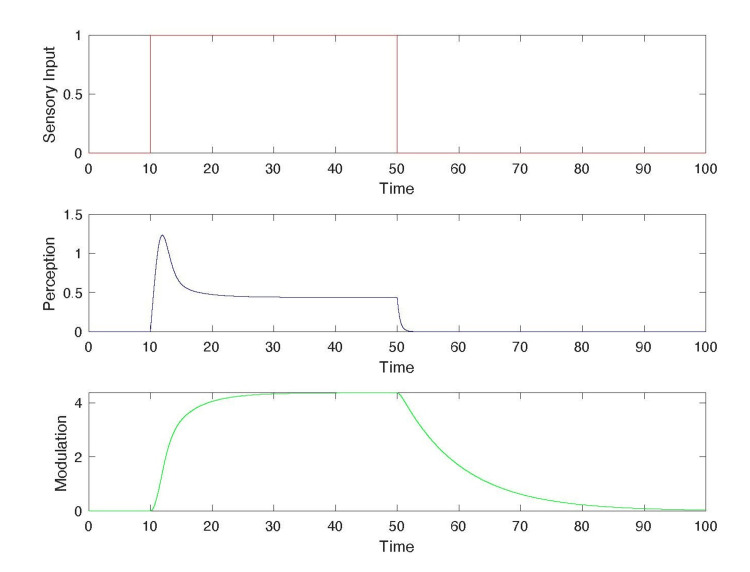
Control-system model of perception and modulation at a stimulus input magnitude of 1 AU. Pain perception *P(t)* (middle tracing, blue) and pain modulation *M(t)* (bottom tracing, green) with Lotka-Volterra-style coupling in response to the pain stimulus time profile *S(t)* (top tracing, red), where the input magnitude is 1 AU (see y-axis). Because of modulation *M(t)*, pain perception *P(t)* registers as a brief spike and then saturates or plateaus at a lower level. The underlying parameter values for the simulation are: Sₘₐₓ = 1, εₚ = 1, βₚ = 0.1, αₚ = 0.5, εₘ = 1, and βₘ = 0.1. The sensory input/pain stimulus onset time point is 10, and the turn-off time point is 50, both in arbitrary time units. Reproduced from Fink W and Raffa RB (2026) [26]. AU: Arbitrary units.

**Figure 2:**
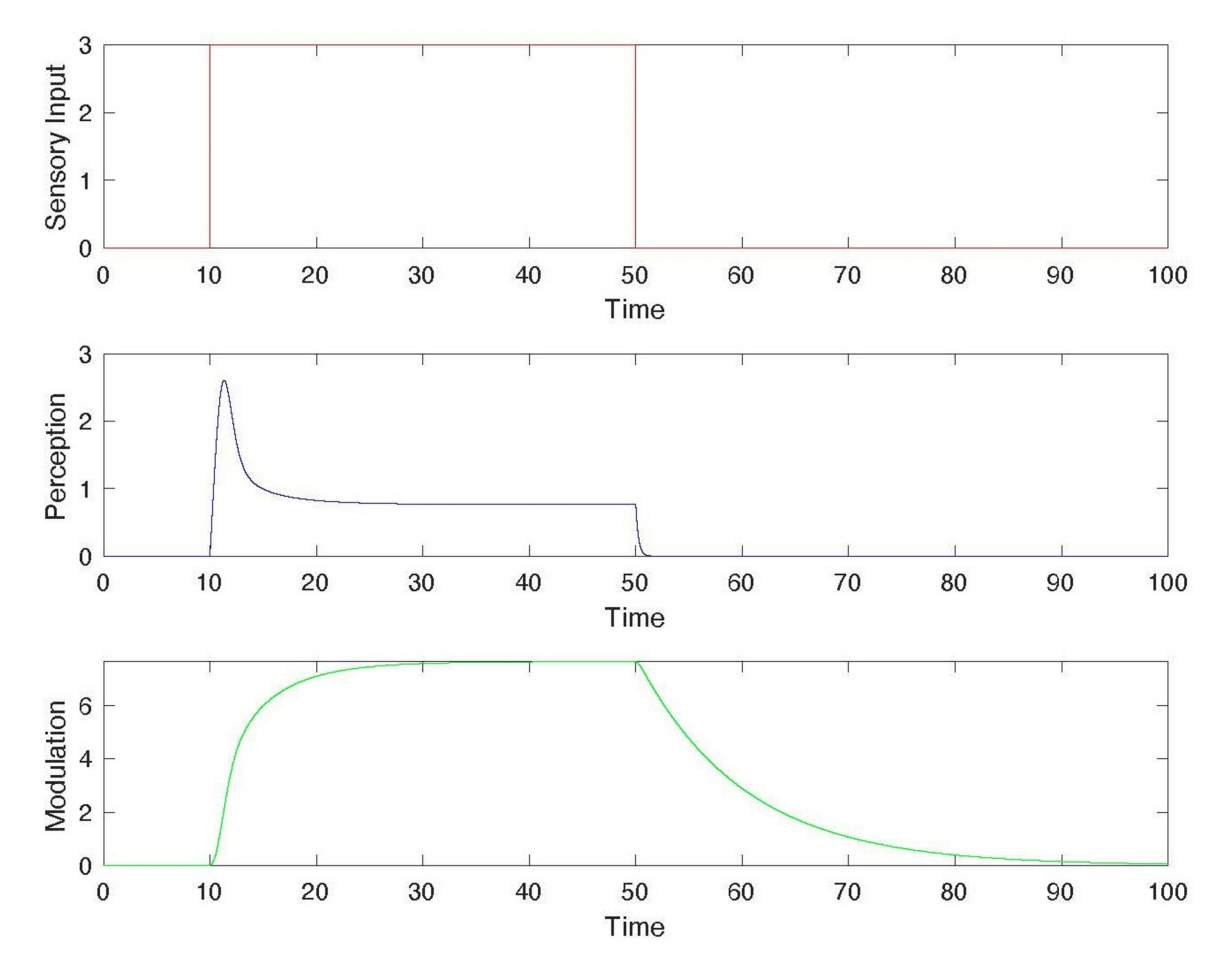
Control-system model of perception and modulation at a stimulus input magnitude of 3 AU. Same as Figure 1, except the stimulus input magnitude Sₘₐₓ is 3 AU. AU: Arbitrary units.

**Figure 3:**
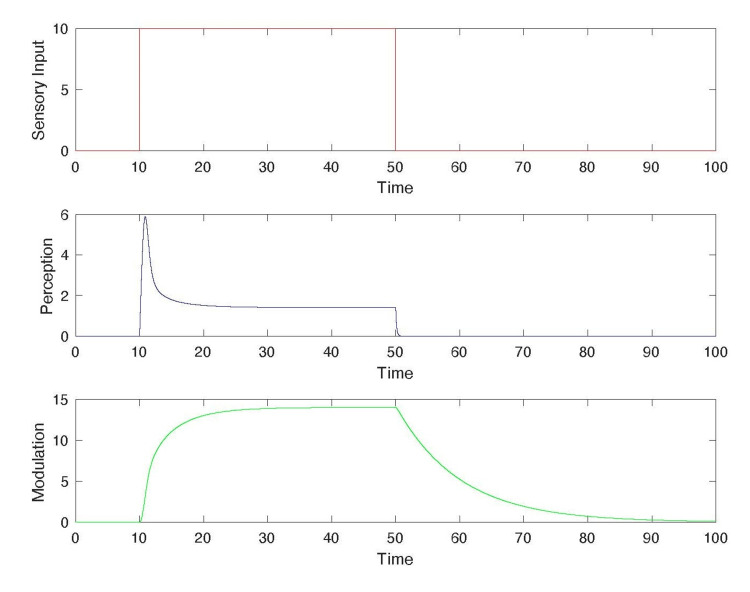
Control-system model of perception and modulation at a stimulus input magnitude of 10 AU. Same as Figure 1, except the stimulus input magnitude Sₘₐₓ is 10 AU. AU: Arbitrary units.

**Figure 4:**
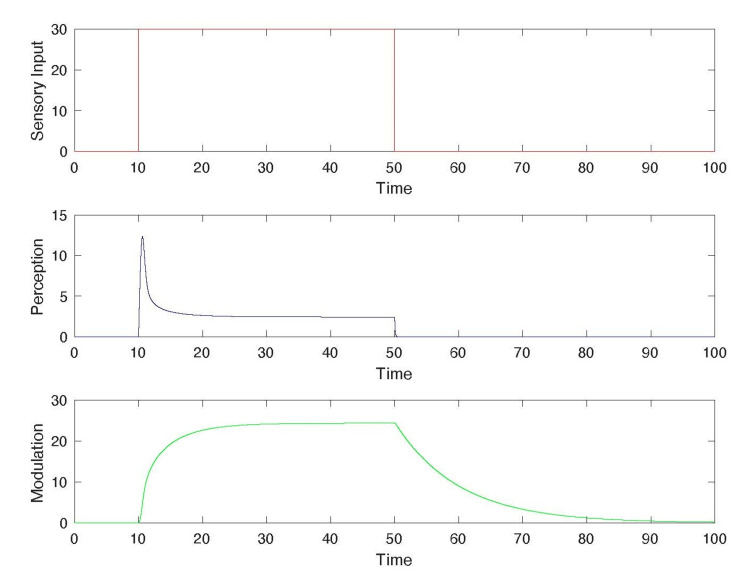
Control-system model of perception and modulation at a stimulus input magnitude of 30 AU. Same as Figure 1, except the stimulus input magnitude Sₘₐₓ is 30 AU. AU: Arbitrary units.

**Figure 5:**
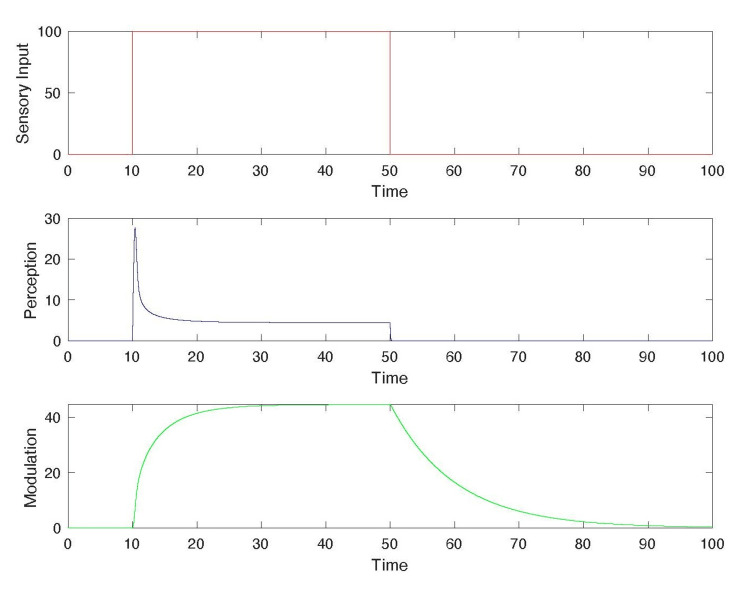
Control-system model of perception and modulation at a stimulus input magnitude of 100 AU. Same as Figure 1, except the stimulus input magnitude Sₘₐₓ is 100 AU. AU: Arbitrary units.

Given the above equations from Fink W and Raffa RB [23,26], Figures 1-5 show the simulation results for the time development of pain perception *P(t)* and modulation *M(t)* using the above model for sensory inputs of pain stimuli *S(t)*, spanning a relative range of 1- to 100-fold in pain stimulus magnitude in arbitrary units (AUs), shown on the y-axis in Figures 1-5. For complete reproducibility, the underlying parameter values for all five simulations are: Sₘₐₓ = 1, 3, 10, 30, 100, εₚ = 1, βₚ = 0.1, αₚ = 0.5, εₘ = 1, and βₘ = 0.1 (see also the legend of Figure 1). Moreover, the initial conditions for all five simulations are: “no pain stimulus present,” i.e., *S(t *= 0) = 0; “no pain perceived,” i.e., *P(t* = 0) = 0; and “no modulation active,” i.e., *M(t* = 0) = 0, representing a pain-free state of the system. The numerical results of the simulations are summarized in Table 1. In each case, the pain input was initiated at tₒₙₛₑₜ = 10 AU, where time is represented as AU for this generalized application, remained constant from t = 10 to 50 AU, and returned to 0 at t_off_ = 50 AU. Modulation began at tₒₙₛₑₜ = 10 AU and continued to change after t_off_ = 50 AU according to the model dynamics. Although the time axis could be interpreted in seconds, the term “arbitrary units” (AU) is used here because it leaves open the duration of the pain signal event, potentially ranging from fractions of a second in the case of a swipe of an animal’s claw, to several seconds in the case of a persistent/prolonged animal bite, or to much longer pain signal events.

**Table 1:** Values related to perception and modulation for each stimulus input. *Maximum perception; **plateau perception, determined at the cut-off of the stimulus; ***maximum modulation. All values are in arbitrary units, as described in the text.

Stimulus	P_max_*	Time to P_max_	P_plateau_**	M_max_***
1	1.2	2	0.4	4.4
3	2.6	1.3	0.8	7.6
10	5.9	0.9	1.4	14
30	12.3	0.6	2.4	24.4
100	27.7	0.4	4.5	44.6

**Figure 6:**
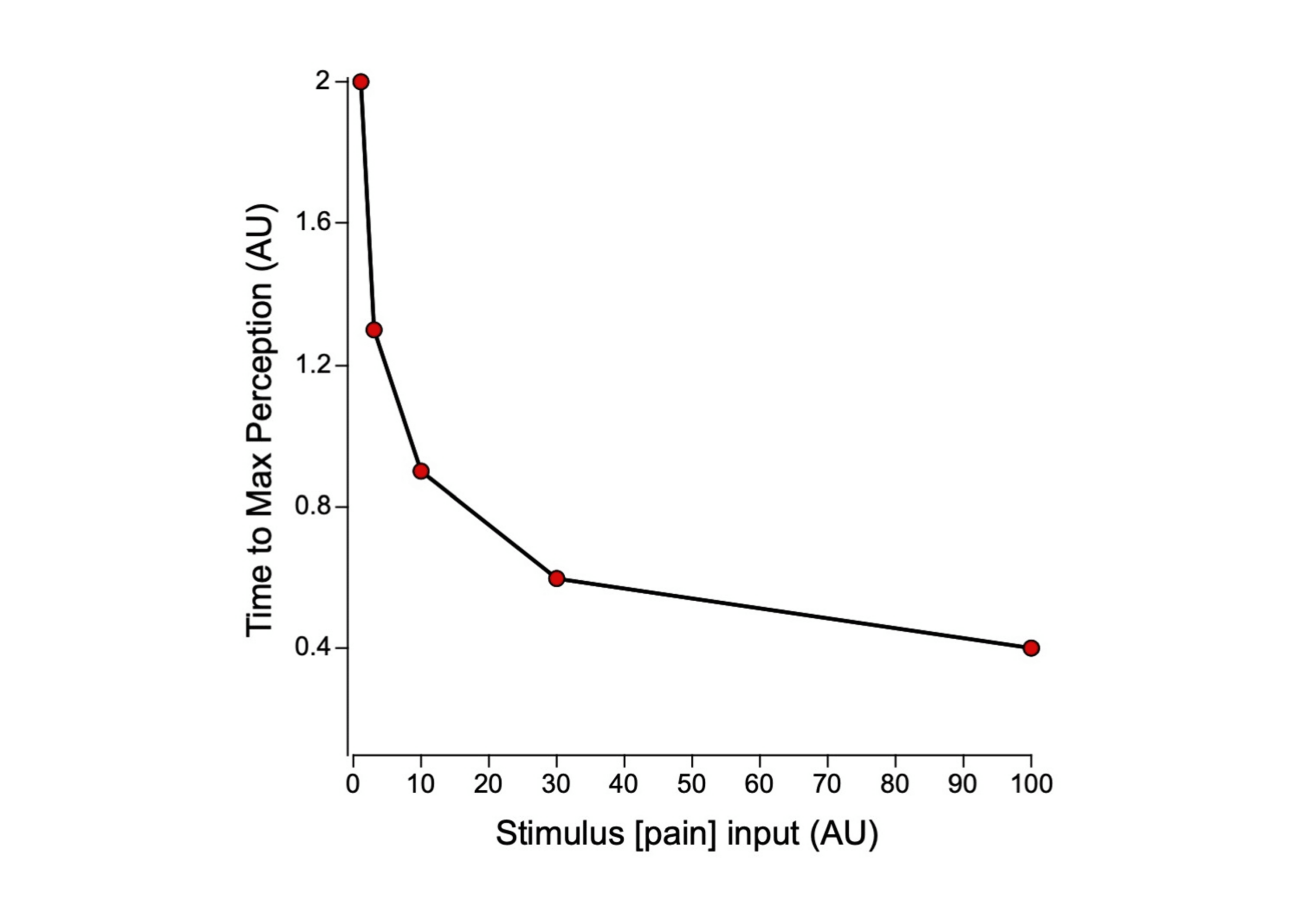
Time to maximum pain perception as a function of pain stimulus input. Relationship between pain stimulus input and the time required to reach maximum pain perception, based on data from Table 1. AU: Arbitrary units.

As seen in each of the five cases, perception increased rapidly in response to the rapid/sudden rise in sensory input. The rate of onset of the rise in perception increased with the magnitude of the stimulus input. Correspondingly, the time to maximum perception decreased with an increase in stimulus intensity (Figure 6).

**Figure 7:**
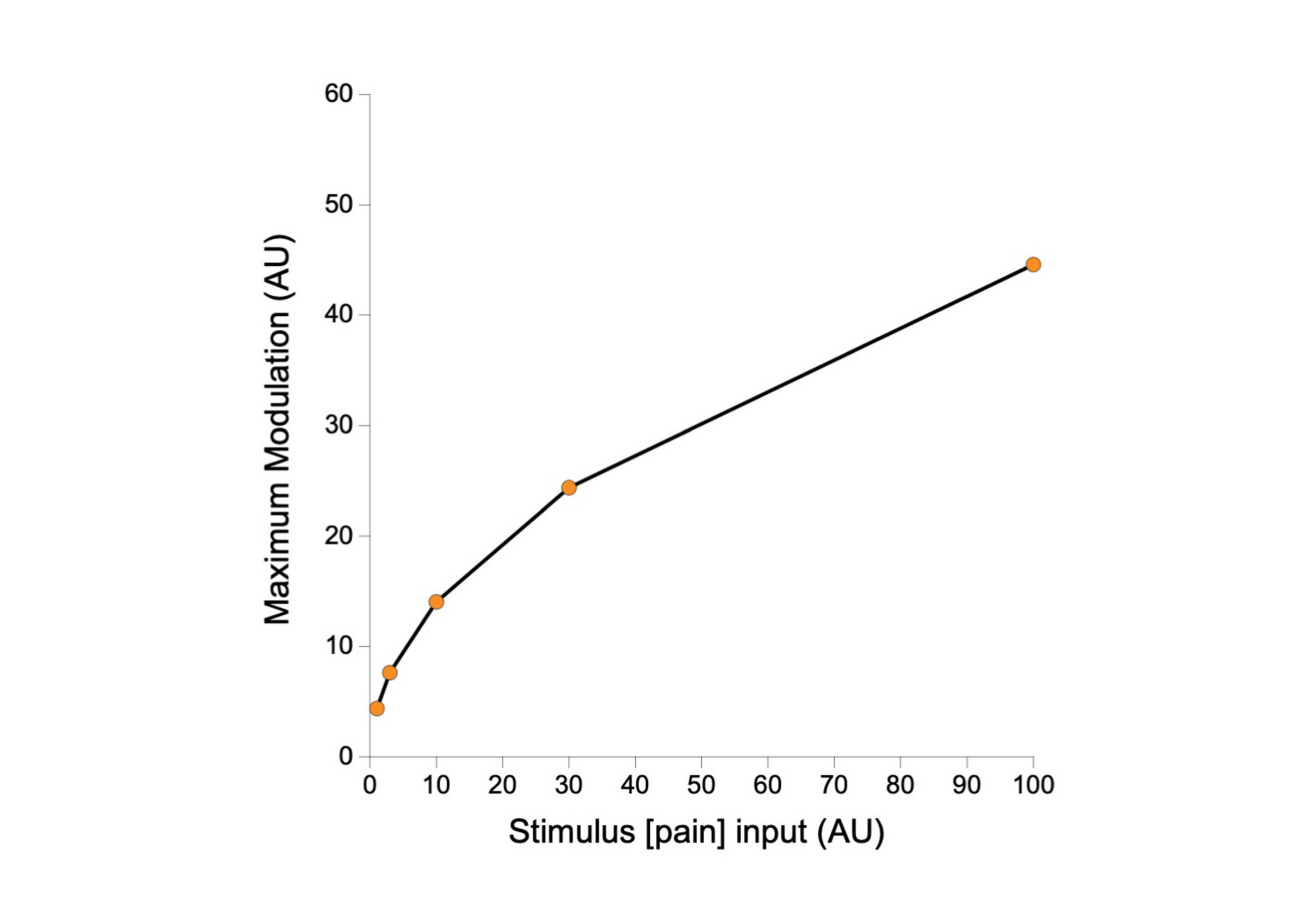
Time to maximum modulation as a function of pain stimulus input. Relationship between pain stimulus input and the time required to reach maximum modulation, based on data from Table 1. AU: Arbitrary units.

The fact that perception did not remain at peak value for the duration of the stimulus input is due to the influence of modulation. As expected, descending inhibitory modulation increased with stimulus intensity (Figure 7), as did the peak magnitude of perception.

As a net result of increasing perception magnitude and increasing inhibitory modulation, perception rises rapidly as a function of stimulus intensity and then begins to decline. However, it does not return to zero. Instead, it saturates or plateaus at a value that is intermediate between zero and maximum perception, at a level that is related to the stimulus intensity.

**Figure 8:**
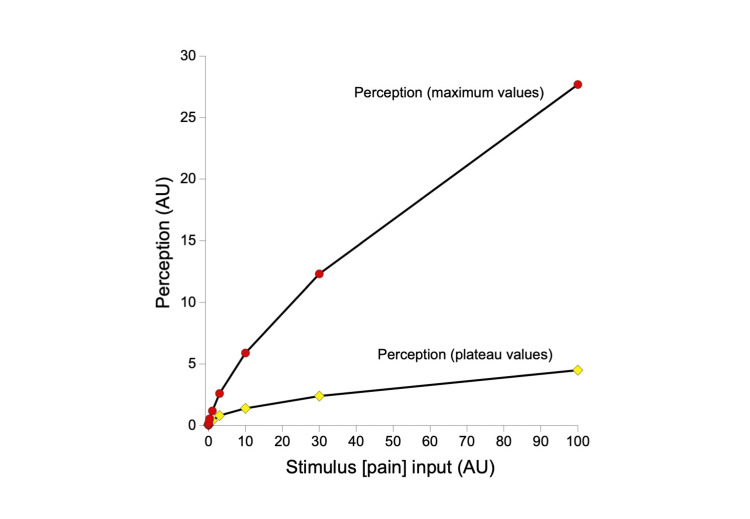
Pain perception, maximum and plateau values, as a function of pain stimulus input. Relationship between pain stimulus input and peak/maximum pain perception (circles) and plateau pain perception (diamonds), based on data from Table 1. AU: Arbitrary units.

The overall relationship between stimulus intensity and perception, taking into account modulation, is shown in Figure 8.

## Discussion

The fight-or-flight response is an inherently complex physiological phenomenon [27-29]. Conceptually, however, it is relatively straightforward: maximization of the likelihood of survival by successfully fighting or fleeing an immediate threat. The participation and internal redirection of physiological processes in the autonomic nervous system are well known and well adapted for this purpose. However, an accurate assessment of whether to persist in the fight-or-flight decision requires additional input, namely, how much damage is being exacted by the threat. This information is provided by pain perception processes [5]. This is somewhat challenging, however: the absence of pain sensation deprives the body of necessary alerts about acute or evolving tissue damage, resulting in significant injury and possible death; excessive or excessively prolonged pain sensation, on the other hand, could distract from the evolving fight-or-flight decision and lead to negative outcomes as well. Thus, ideally, pain should be perceived but not eliminated, which is a difficult challenge. A coupled feedback control loop, comprising, for example, ascending and descending pathways, can accomplish this goal [23,24]. This broad concept is often stated in qualitative terms, but a more formal, i.e., mathematical, model, such as [23], could provide insight into the functioning of this system, informing the potential design of better-suited analgesics, namely, those that target the system rather than individual components.

The concept of using control systems theory analysis for practical clinical application was proposed by us, to our knowledge, for the first time and was recently modeled [23,24]. The work described here attempts to substantiate a qualitative → quantitative foundation of our system-theoretical model of the ascending and descending pain pathways [23,24]. In this first application to the fight-or-flight response, the input stimulus was an acute-onset, constant pain stimulus of finite duration, analogous to a swipe by an animal’s claw. The perception, *P(t)*, of such an injurious stimulus is temporally and quantitatively related to the magnitude of the pain stimulus, i.e., monitoring, as a result of the underlying system-theoretical model. That is, the rate of onset of pain perception and the magnitude of perception increased as the magnitude of the acute pain stimulus increased. Thus, information regarding the nature of the first incurred injury and its threat to survival can be assessed as it occurs. At all levels of pain stimulus, pain perception is transient, which also favors survival, because a single injury might not warrant capitulation, whereas multiple injuries might. The driving factor that explains the transient nature of the acute response is the modulation, i.e., damping, *M(t)*, of the afferent input, the magnitude of which also increases with the magnitude of the pain stimulus and the perception of it. This adaptation ensures that a single stimulus does not persist and prevent detection of a subsequent signal, analogous to a radar screen being “cleared” in order to detect the next signal.

## Conclusions

In order to be useful, the perception of pain needs to be a reliable indicator of the actual magnitude and duration of the pain sensory input, i.e., damage, so that appropriate action can be taken, such as fight or flight, but not so large or persistent as to be a debilitating distraction and thereby an impediment to health or survival.

We used Lotka-Volterra-style coupling to model ascending and descending modulation pain pathways as a coupled control and feedback loop. Here, we applied the model to examine the influence of modulation on the pain stimulus signal and found separate response functions that allow for both a quantitative assessment of immediate threat and damping of the signal in order to preclude sensory overload that might lead to counterproductive inaction. Although this work is an initial conceptual exploration rather than a demonstration of applied utility, we found that peak immediate perception may serve as a quantitative indicator of the immediate threat, i.e., injury level. Finally, plateau pain perception is less than peak pain perception for an individual pain input, thus preventing obscuration of subsequent new acute, i.e., cumulative, injury. Since the study is based on mathematical modeling without experimental or physiological validation as yet, further experimental or clinical studies will be necessary to validate the proposed mechanisms and strengthen the applicability of the conclusions.
